# Epigenetic suppression of E-cadherin expression by Snail2 during the metastasis of colorectal cancer

**DOI:** 10.1186/s13148-018-0592-y

**Published:** 2018-12-12

**Authors:** Yue Hu, Mingrui Dai, Yayuan Zheng, Jiaxin Wu, Bin Yu, Haihong Zhang, Wei Kong, Hui Wu, Xianghui Yu

**Affiliations:** 1 National Engineering Laboratory for AIDS Vaccine, School of Life Sciences, Jilin University, Changchun, Jilin province People’s Republic of China; 2 Key Laboratory for Molecular Enzymology and Engineering, the Ministry of Education, School of Life Sciences, Jilin University, Changchun, Jilin province, People’s Republic of China

**Keywords:** Colorectal cancer, Snail2, E-cadherin, Metastasis, EZH2, HDAC6

## Abstract

**Background:**

The transcription factor Snail2 is a repressor of E-cadherin expression during carcinogenesis; however, the specific mechanisms involved in this process in human colorectal cancer (CRC) remain largely unknown.

**Method:**

We checked the expression of Snail2 in several clinical CRC specimens. Then, we established Snail2-overexpressing and knockdown cell lines to determine the function of Snail2 during EMT and metastasis processes in CRC. In addition, we used luciferase reporter assay to explore how Snail2 inhibits the expression of E-cadherin and induces EMT.

**Results:**

We found that the expression of Snail2 was higher in clinical specimens of colorectal cancer (CRC) compared to non-cancerous tissues. Overexpression of Snail2 induced migration and metastatic properties in CRC cells in vitro and in vivo. Furthermore, overexpression of Snail2 promoted the occurrence of the epithelial–mesenchymal transition (EMT), downregulating the expression of E-cadherin and upregulating that of vimentin. Specifically, Snail2 could interact with HDAC6 and then recruited HDAC6 and PRC2 to the promoter of E-cadherin and thus inhibited the expression of E-cadherin, promoting EMT and inducing invasion and metastasis of CRC.

**Conclusion:**

Our study reveals that Snail2 might epigenetically suppress the expression of E-cadherin during CRC metastasis.

## Introduction

Colorectal cancer (CRC) is the third most common cancer and the fourth most common cause of death worldwide [1]. The past decade has witnessed important advances in CRC treatment, such as the development of effective anticancer drugs, including angiogenesis inhibitors and antibodies targeting cancer-associated proteins. However, the prognosis of patients with CRC remains poor [2]. Unfortunately, a significant number of patients with colorectal carcinoma who undergo a supposedly curative operation develop local recurrence or distant metastasis leading to shorter survival. Thus, preventing metastasis is fundamental to treat colorectal cancer. Metastasis is closely related to the epithelial–mesenchymal transition (EMT), a process in which cells gradually lose their epithelial phenotype and obtain a mesenchymal phenotype [3]. Numerous studies have shown that EMT is one of the important steps in the progression of tumors and is a necessary condition for the increased invasive ability of tumor cells in the progression of breast [4, 5], prostate [6], liver [7], and lung cancer [8]. Cancer cells undergoing EMT exhibit both morphological changes and molecular alterations, as demonstrated by the decreased expression of epithelial markers, including E-cadherin and β-catenin, and increased expression of mesenchymal markers, such as N-cadherin and vimentin.

Therefore, the regulation of E-cadherin plays an essential role in tumor progression. The regulation of EMT involves multiple growth factors (TGF, HGF, EGF) [9] and transcriptional inhibitors (Snail, ZEB1, twist) [10]. Snail is the first discovered transcriptional inhibitor of E-cadherin. The Snail protein family includes Snail1, Snail2 (SLUG), and Snail3 (Smuc). Snail proteins specifically bind to the E-box element in the E-cadherin gene through their zinc finger domain. A study has shown that the transcriptional repressor Slug promotes tumor invasion and predicts the outcome in patients with lung adenocarcinoma [11]. Another study has reported that Snail1 combines competitive displacement of ASCL2 and epigenetic mechanisms to rapidly silence the EPHB3 tumor suppressor in CRC [12]. Moreover, it has been reported that Snail1 interacts with G9a to mediate E-cadherin repression in human breast cancer [13]. Although several studies have reported that NANOG and HMGA2 could regulate Snail2 to affect tumor invasion and metastasis [14, 15], few studies have investigated the relationship between Snail2 and EMT in CRC, and its implications in tumor progression.

It is known that gene expression and the activation of signaling pathways are both subjected to epigenetic regulation. Many histone methyltransferases such as EZH2 and G9a have been reported to participate in cancer metastasis. EZH2 is the catalytic core subunit of polycomb repressive complex 2 (PRC2), a complex that also includes embryonic ectoderm development (EED) and suppressor of zeste 12 (SUZ12) [16]. PRC2 methylates lysine 27 of histone H3 (H3K27) and lysine 9 of histone H3 (H3K9) to promote transcriptional silencing [16]. EZH2 has emerged as a crucial regulator of wound healing [17], tumorigenesis [18], fibrogenesis [19], and EMT [20]. Additionally, a study has shown that EZH2 induces EMT in endometriosis. Mechanistically, EZH2 induces EMT by repressing the expression of E-cadherin through histone H3K27 trimethylation of the E-cadherin gene in prostate and breast cancer [20]. Histone deacetylases have also been shown to be involved in the metastatic process of cancer, and some HDAC inhibitors have been used in the treatment of certain cancers. The altered activity of HDACs can result in the deregulation of important biological processes including cell proliferation, differentiation, apoptosis, and migration [21]. It has been found that the inhibition of HDACs promotes epithelial gene expression in pancreatic cancer cells and prevents tumor metastasis. Meanwhile, in leukemia and prostatic cancer cells, HDAC1, HDAC2, and HDAC3 inhibitors display different antitumor activity depending on their p53 status [22]. However, the role of EZH2 and HDACs in the metastasis of CRC cells remains unclear.

In the present study, we found that Snail2 is highly expressed in patients with CRC and that it successfully promotes the migration and invasion of CRC cells both in vitro and in a mouse model. Additionally, the overexpression of Snail2 in CRC cells leads to the transition from epithelial to mesenchymal phenotype in these cells. Notably, EZH2 and HDACs enhance the suppression of E-cadherin caused by Snail2, facilitating EMT and metastasis of CSC cells in vitro. Thus, Snail2 might epigenetically suppress the expression of E-cadherin during the metastasis of CRC.

## Methods

### Tissue samples

Seventeen paired CRC tissues and corresponding adjacent non-cancerous tissues were obtained from patients who underwent surgical treatment at the China-Japan Union Hospital of Jilin University, China. All non-cancerous colon samples were obtained from tissues adjacent to, but separate from, the tumors. The use of human specimens in this study was approved by the Ethics Committee at Jilin University. Written informed consent was provided by each patient. Tissue samples were immediately frozen in liquid nitrogen and stored at − 80 °C until use.

### Cell culture and transfection

SW480 cells and SW620 cells (CCL-228, CCL-227, ATCC) were cultured in RPMI1640 (Sigma-Aldrich, USA) supplemented with 10% FBS (Invitrogen, USA) in a humidified atmosphere and 5% CO_2_, at 37 °C. SW480 cells were infected with a lentivirus expressing EGFP and Snail2 or their control lentivirus (Hanbio, China) at an MOI of 10. SW620 cells were infected with a lentivirus expressing EGFP and Sh-Snail2 or their control lentivirus (Hanbio, China) at an MOI of 20. Stable cell lines were selected using puromycin (1 mg/mL, INALCO, USA). ShRNA(Snail2) sequence 5′-GATGCATATTCGGACCCACACATTA-3′.

### Western blotting and antibodies

Protein samples were mixed with 4× SDS loading buffer. Samples were boiled for 5 min at 98 °C, and proteins were separated by Biofuraw™ Precast Gel (180-8008H, Tanon, China) and transferred to PVDF membranes, and then, the following primary antibodies and dilutions were used: Snail2 monoclonal antibody (sc-166467, 1:100, Santa Cruz, USA), E-cadherin polyclonal antibody, vimentin polyclonal antibody, fibronectin polyclonal antibody, and GAPDH polyclonal antibody (20874-1-AP, 15613-1-AP, 10366-1-AP, 60004-1-AP, 1:1000, Proteintech, China). Secondary antibodies were anti-rabbit or anti-mouse HRP-conjugated IgG (SA00001-4, 1:2000, Proteintech, China). Membranes were incubated with 1 mL ECL western blotting substrate (Promega, USA) for 1 min at room temperature and then exposed to x-ray film.

### Quantitative real-time PCR

Total RNA from tissue samples was isolated with TRIzol (Invitrogen, USA). qPCR was performed in the CFX96 Real-Time PCR Detection System (Bio-Rad, USA) using TransStart Top Green qPCR SuperMix (Transgene, China) according to the manufacturer’s instructions. The primers used for PCR amplification were as follows: E-cadherin forward, 5′-TGCCCAGAAAATGAAAAAGG-3′ and reverse, 5′-GTGTATGTGGCAATGCGTTC-3′; fibronectin forward, 5′-CAGTGGGAGACCTCGAGAAG-3′ and reverse, 5′-TCCCTCGGAACATCAGAAAC-3′; vimentin, forward, 5′-GAGAACTTTGCCGTTGAAGC-3′ and reverse, 5′-GCTTCCTGTAGGTGGCAATC-3′; actin forward, 5′-CGTGGACATCCGCAAAGACC-3′ and reverse, 5′-CGTGGACATCCGCAAAGACC-3′; and Snail2 forward, 5′-ATACCACAACCAGAGATCCTCA-3′ and reverse, 5′-GACTCACTCGCCCCAAAGATG-3′. qPCR data were analyzed using the comparative Ct method, and the expression of target genes was normalized to that of β-actin.

### Wound healing assays

Wound healing assays were performed as described previously [23, 24]. Cells were seeded in six-well plates (1 × 10^5^ cells/well) and cultured at 37 °C. After 24 h, a pipette tip was used to create a wound in the cell monolayer. The cells were then cultured in RPMI-1640 supplemented with 2% FBS at 37 °C. Inhibitors, if used, were added to the complete medium. The width of the wound was measured under a microscope (Nikon, Japan) 24 h after the scratch.

### Invasion assays

Invasion assays were performed as described previously [25, 26]. Boyden chambers were coated with Matrigel (BD Biosciences, USA). According to the manufacturer’s protocol, cells (5 × 10^3^) were seeded on Matrigel in the upper chamber, and the bottom chamber was filled with culture medium containing LPA (10 mM) as chemoattractant. Cells that invaded through the Matrigel-coated membrane after 24 h were fixed with paraformaldehyde and stained with crystal violet. The fold change in invasion was calculated by dividing the number of cells in SW480-Snail2 by the number of cells in the control cells. All experiments were conducted at least twice in triplicate.

### Liver metastasis model

Female BALB/c nude mice (6–8 weeks old) were purchased from Charles River (China). All animal studies were conducted in accordance with legal and institutional guidelines. The procedures were approved by the Ethical Committee of Care and Use of Laboratory Animals at Jilin University. Mice were injected with SW480-N (control) or SW480-Snail2 cells (5 × 10^5^ cells/mouse) via subcutaneous injections (eight mice/group). Tumor growth was measured every 3 days with a caliper, and the tumor volume was calculated with the formula: (length × width^2^)/2 (mm^3^). Visible lung metastatic nodules were examined macroscopically or embedded in paraffin, sectioned, and subjected to H&E and immunohistochemical staining.

### H&E and immunohistochemistry

The experiment was performed as described previously [27]. After mice were sacrificed, livers were excised, fixed in 4% paraformaldehyde, and then embedded in paraffin. After blocking with 5% normal goat serum, the sections were incubated with an anti-Ki-67 monoclonal antibody (1:200, Abcam, USA) at 4 °C overnight. The sections were then washed with PBS, incubated with horseradish peroxidase (HRP)-labeled sheep anti-mouse secondary antibody, and then reacted with the chromogen diaminobenzidine (DAB).

### Luciferase assay

For luciferase assays, the Dual-Luciferase Reporter Assay System (Promega, USA) was used as previously described [28]. Briefly, SW480-Snail2 and control cells were co-transfected with a E-cadherin-promoter containing luciferase construct together with a plasmid expressing Renilla luciferase (pGL3-Bisic, Promega). Firefly luciferase activity was normalized to Renilla luciferase activity to control for transfection efficiency. Luminescence measurements were performed using a VICTOR X2 Multilabel Plate Reader (PerkinElmer, USA).

### Chromatin immunoprecipitation (ChIP) assays

Chromatin immunoprecipitation assays were performed according to the protocol described previously [29]. In brief, 1 × 10^7^ of SW480-N or SW480-Snail2 cells grown to 80% of confluence was used for each ChIP. The cells were crosslinked with formaldehyde solution (Sigma, USA) at room temperature. Cell lysates were subjected to sonication and then incubated with antibody overnight, followed by incubation with a 50% slurry of protein G–agarose for 3 h. Bound DNA–protein complexes were eluted, and crosslinks were reversed after a series of washes. Purified DNA was resuspended in TE buffer (10 mM Tris–HCl and 1 mM EDTA (pH 8.0)) for PCR. H3K27me3 (C15410195, 5 μg/ChIP) antibodies was purchased from Diagenode (Belgium). The primers for the E-cadherin promoter were: 5′-GCCCTTTCTGATCCCAGGTC-3′ and 5′-TAGCCTGGAGTTGCTAGGGT-3′.

### Immunoprecipitation

Immunoprecipitation assays were performed according to the protocol described previously [30]. In brief, the plasmids of Snail2-HA, EZH2 and HDAC6 were transfection into SW480 cells. Lysates were applied to anti-HA antibody-conjugated agarose beads (Roche, Switzerland) and washed with washing buffer (20 mM Tris, pH 7.5, with 0.1 M NaCl, 0.1 mM EDTA, 0.05% Tween-20). The beads were eluted with elution buffer (0.1 M glycine, pH 2.0), followed by SDS-PAGE and western blotting.

### Statistical analysis

The GraphPad Prism software for Windows (GraphPad Software, USA) was used for all statistical analyses. The results are expressed as mean values ± SD. Significant differences between two groups were assessed using paired two-tailed Student’s *t* tests. A *P* value < 0.05 was considered statistically significant.

## Results

### Snail2 is overexpressed in CRC tissues

**Fig. 1:**
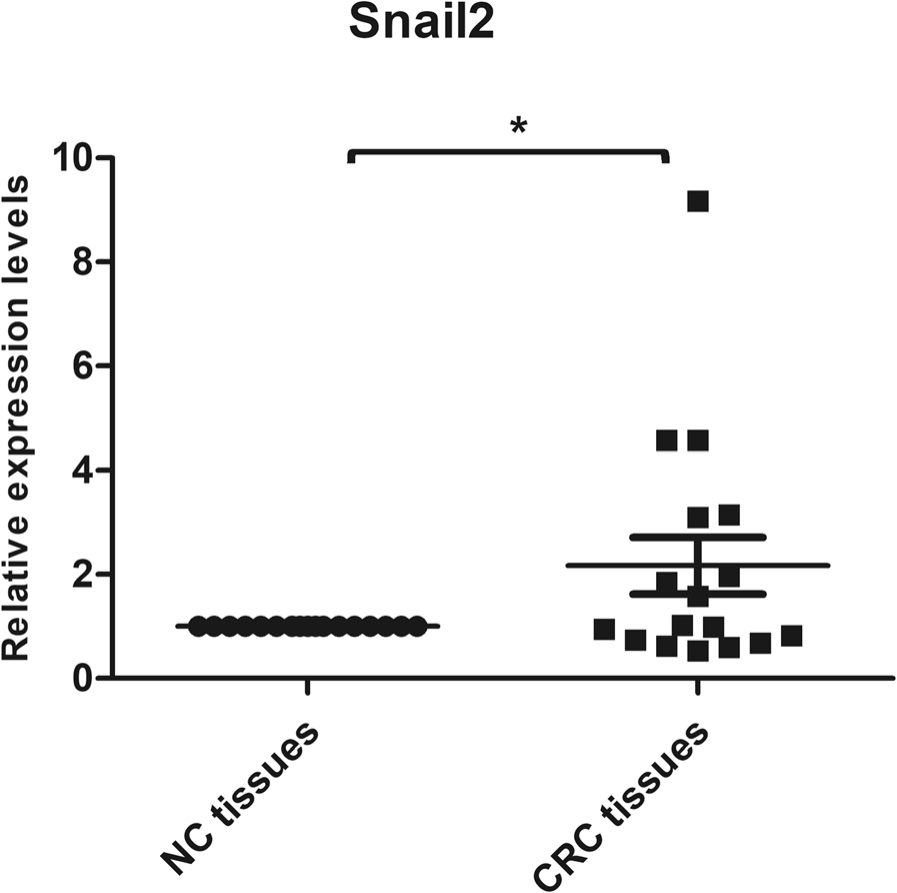
Snail2 is overexpressed in colorectal cancer. The expression of Snail2 in CRC and adjacent non-cancerous tissues were examined by qPCR. Data represent the mean ± SD. *P ≤ 0.05

To check the expression level of Snail2 in CRC, quantitative PCR (qPCR) was performed in 34 specimens: 17 specimens of CRC tissues and 17 of paired adjacent non-cancerous tissues. We found that Snail2 was significantly upregulated in CRC tissues (Fig. 1). According to the information the patients had provided, we classified them as patients with colorectal or colon cancer; because Snail2 was highly expressed in the colon, we decided to focus on colon cancer in the remaining experiments.

### Snail2 promotes migration and metastasis in CRC cells, but does not promote proliferation

**Fig. 2:**
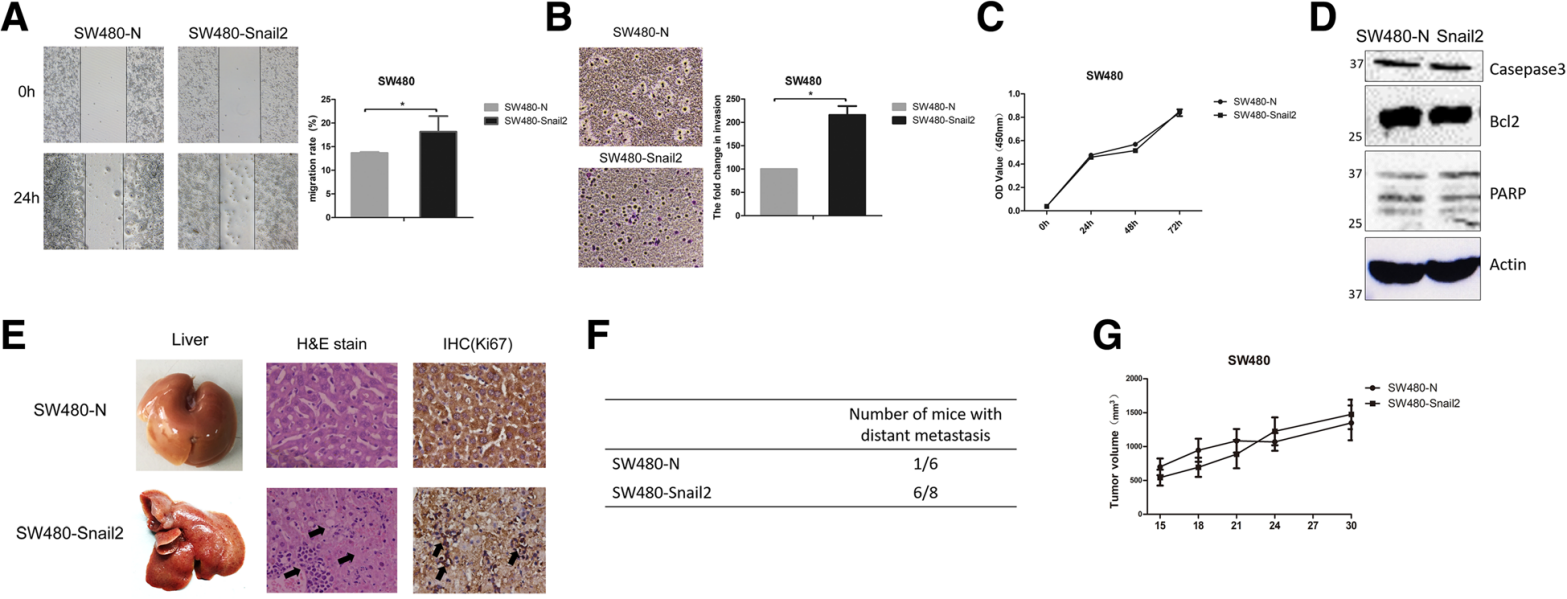
Snail2 induces migration and metastasis in CSC cells, but does not promote cell proliferation. **a** The migration of SW480 cells (overexpressing Snail2 or control) was analyzed by wound healing assays. The statistical analysis is shown in the bar graph (mean ± SD from three independent experiments), and a representative experiment is shown in the right panel. Phase contrast images were taken at × 4 magnification. **b** The invasiveness of SW480 cells (overexpressing Snail2 or control) was analyzed in invasion assays. The fold change in invasion is shown in the bar graph (mean ± SD from three independent experiments), and a representative experiment is shown in the left panel. Phase contrast images were taken at × 10 magnification. **c** Proliferation of SW480-Snail2 and control cells was examined using the CCK-8 assay Kit. **d** Apoptosis analysis, caspase3, Bcl2, PARP, and PARP cleavage was examined in SW480-N and SW480-Snail2 by western blotting. **e** SW480 cells (overexpressing Snail2 or control) were subcutaneously injected into nude mice. Each group had six mice. After 30 days, the mice were sacrificed and the liver was dissected. Liver metastatic nodules were examined macroscopically or paraffin-embedded, cut into sections, and subjected to H&E and immunohistochemical staining. Immunohistochemical staining of Ki-67 expressed in liver tissues. (Arrowheads indicate liver metastases). Phase contrast images were taken at × 40 magnification. **f** The total number of mice with distant metastasis at 30 days after injection of Snail2-overexpressing or control SW480 cells. **g** Weight of tumors following subcutaneous injection of Snail2-overexpressing or control SW480 cells

To test whether Snail2 could regulate the migratory, invasive, and proliferative abilities of CRC cells, we retrovirally established the stable overexpression of Snail2 in SW480 cells. The effect of Snail2 on cell migration was first assessed in wound healing assays. SW480-Snail2 cells had a significantly higher migration rate compared with control cells (SW480-N, Fig. 2a): the migration rate increased to 140%. Moreover, SW480-Snail2 cells showed a greater degree of invasion through Matrigel (Fig. 2b): Snail2 increased the invasive capacity of the cells to 200%. Contrarily, compared with control, SW480-Snail2 cells did not show higher proliferation (Fig. 2c). For apoptosis analysis, caspase3, Bcl2, PARP, and PARP cleavage were examined. There were no differences between SW480-N and SW480-Snail2 cells (Fig. 2d). To confirm our results in vivo, we investigated whether Snail2 can regulate the tumorigenic properties of CRC cells. SW480-Snail2 and control cells were subcutaneously injected into nude mice. Tumor size was measured every week up to 4 weeks. After 30 days, we dissected the mice and found that the liver of the mice injected with SW480-Snail2 had undergone metastases and was necrotic. H&E staining showed irregular liver cell arrangement in the mice injected SW480-Snail2; no contour was detected, and there was no clear distinction between the nucleus and the cytoplasm of the cells. To further confirm these results, we performed immunohistochemical (IHC) analyses for Ki-67 in the liver tissues of mice injected SW480-Snail2 and control. The result revealed that liver tissues of the mice injected SW480-Snail2 correlated with high expression of Ki67. Additionally, we identified the presence of eosinophilic bodies in the cells. Furthermore, large areas of necrosis, including bridging, zonal, and focal necrosis were evident (Fig. 2e, f). As already observed in vitro, Snail2 overexpression did not significantly induce proliferation of the cells (Fig. 2g). Therefore, Snail2 significantly increases the invasive ability of CRC cells in vitro and in vivo.

### Snail2 induces EMT in CRC cells

**Fig. 3:**
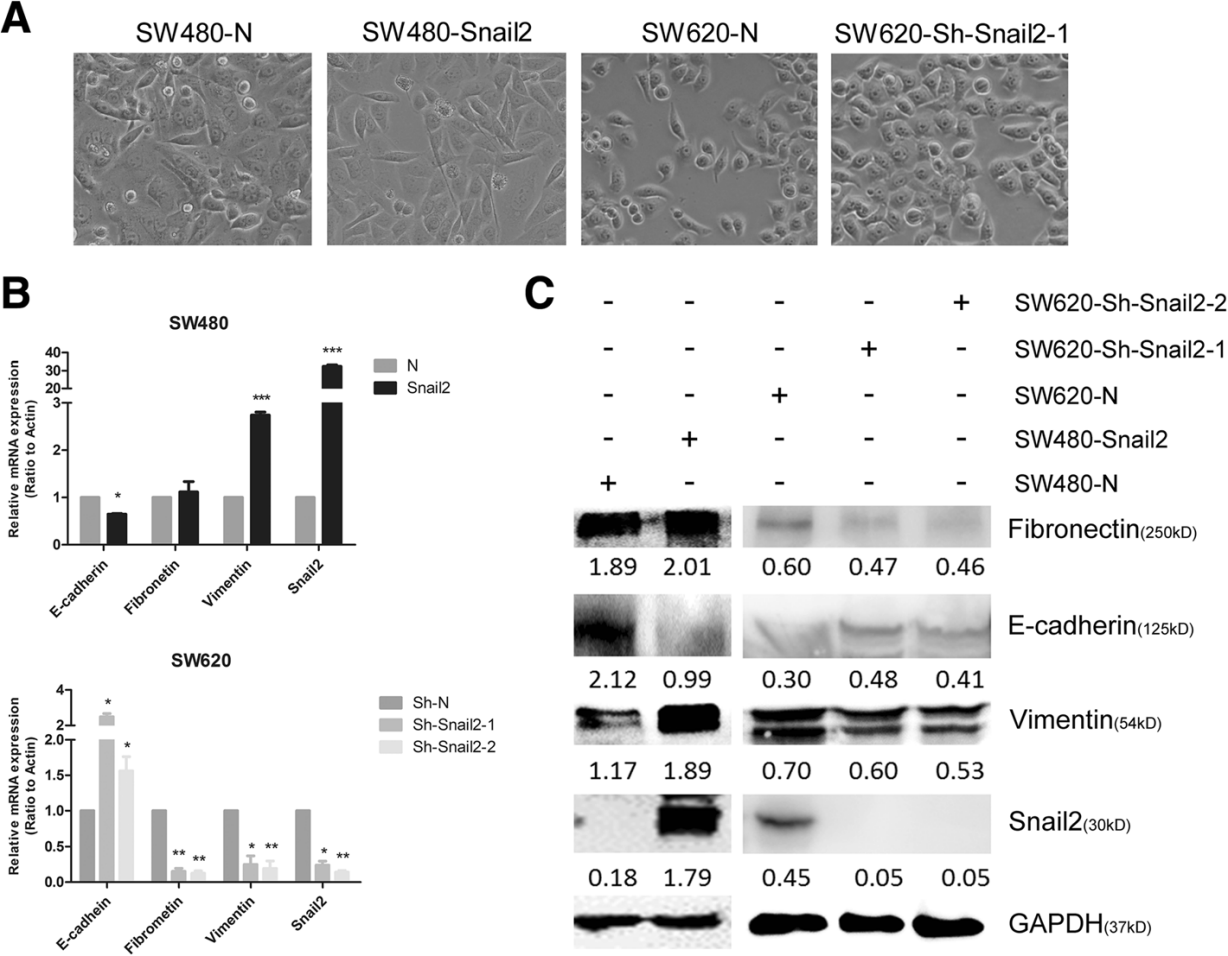
Snail2 regulates EMT in CRC cells. **a** Morphological changes associated with EMT as observed in phase contrast images of SW480-N, SW480-Snail2, SW620-N, and SW620-Sh-Snail2 cells. Phase contrast images were taken at × 10 magnification. **b** Expression of E-cadherin, fibronectin, vimentin, and Snail2 in SW480-N, SW480-Snail2, SW620-N, and SW620-Sh-Snail2 cells was analyzed by qPCR and shown as “relative mRNA levels.” Data represent the mean ± SD. *P ≤ 0.05; **P ≤ 0.01; ***P ≤ 0.001. **c** Expression of E-cadherin, fibronectin, vimentin, and Snail2 in these cells was analyzed by western blotting

During establishment of the Snail2-overexpressing and knockdown cell lines, we observed that the cells exhibited a fibroblast-like morphology compared with their respective control cells, which had an epithelial phenotype (Fig. 3a). The morphology of cell lines with lower expression of Snail2 (SW480-N, SW620-Sh-Snail2) was tightly connected, and circular or diamond shaped, whereas SW480-Snail2 and SW620-N cells were scattered, with reduced cell connections, and fusiform. This observation was further confirmed by qPCR and western blot analyses of epithelial and mesenchymal molecular marker expression; Snail2 overexpression decreased the expression of E-cadherin, an epithelial marker, and it increased the expression of vimentin, a mesenchymal marker. On the contrary, silencing of Snail2 increased the expression of E-cadherin, an epithelial marker, and decreased the expression of fibronectin, vimentin, mesenchymal markers (Fig. 3b, c). These data suggest that Snail2 regulates EMT in CRC cells.

### Snail2 may repress E-cadherin expression through histone methylation and deacetylation

To explore how Snail2 inhibits the expression of E-cadherin and induces EMT, the E-cadherin promoter region was sub-cloned into the pGL3-basic luciferase vector (to obtain the pGL3-E-cadherin-Luc vector) and the effect of Snail2 on E-cadherin transcription was estimated upon transfection of pGL3-E-cadherin-Luc in SW480-Snail2 and control cells. Transfection of pGL3-E-cadherin-Luc in SW480-Snail2 decreased the luciferase activity, indicating that Snail2 inhibits the expression of E-cadherin.

**Fig. 4:**
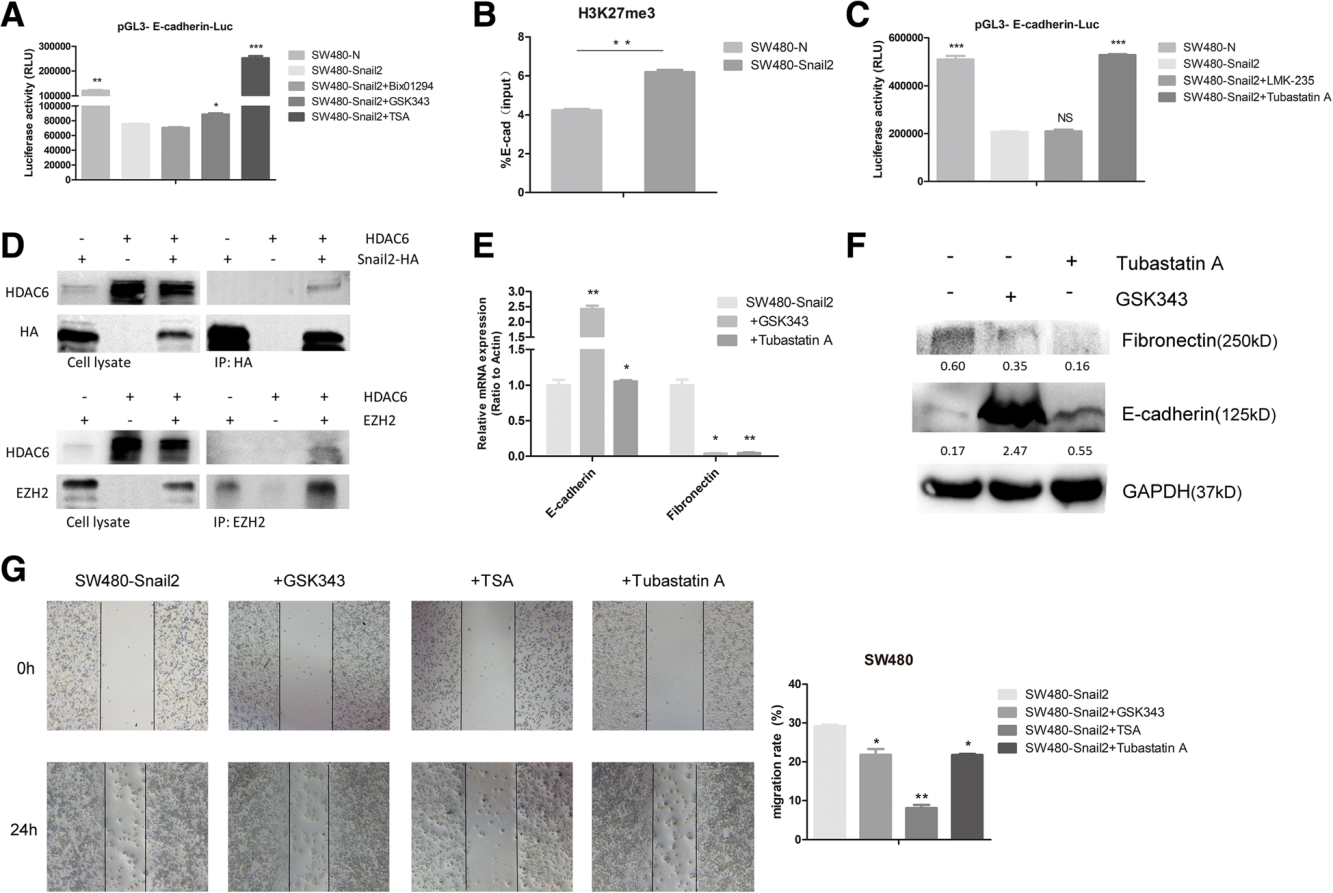
Snail2 represses E-cadherin expression through histone methylation and deacetylation. **a** SW480-N and SW480-Snail2 cells express the luciferase reporter plasmid pGL3-E-cadherin-Luc. Bix01294, GSK343, and TSA were added to the cell growth medium and 24 h later, the luciferase activity was assayed and normalized to that of Renilla (pRL-SV40), which served as an internal control. Each data point represents the mean ± SD. *P ≤ 0.05; **P ≤ 0.01; ***P ≤ 0.001. Experiments were performed twice in triplicate. **b** The level of H3K27me3 at the E-cadherin promoter in SW480-N and SW480-Snail2 was analyzed by ChIP. ChIP samples were also analyzed by quantitative real-time PCR (mean ± SD from three separate experiments; bottom panel). **c** LMK-235 and Tubastatin A were added to the cell’s growth medium, and 24 h later, the luciferase activity was assayed and normalized to that of Renilla (pRL-SV40), which served as an internal control. Each data point represents the mean ± SD. *P ≤ 0.05; **P ≤ 0.01; ***P ≤ 0.001. Experiments were performed twice in triplicate. **d** Co-immunoprecipitation assays were performed in SW480 cells transiently co-expressing plasmids encoding HA-tagged Snail2 and HDAC6 (upper), or HDAC6 and EZH2 (lower). Cell extracts were immunoprecipitated with HA antibodies or EZH2 antibodies, and bound HDAC6 was examined by western blotting. **e** GSK343 and TSA were added to the cell growth medium in SW480-Snail2 cell line and 48 h later, expression of E-cadherin and fibronectin in cells was analyzed by qPCR and shown as “relative mRNA levels.” Data represent the mean ± SD. *P ≤ 0.05; **P ≤ 0.01; ***P ≤ 0.001. **f** GSK343 and TSA were added to the cell growth medium in SW480-Snail2 cell line and 48 h later, expression of E-cadherin and fibronectin in cells was analyzed by western blotting. **g** The migratory ability of SW480-Snail2 cells treated or not with GSK 343, TSA, and Tubastatin A for 24 h was analyzed in wound healing assays. The statistical analysis is shown in the bar graph (mean ± SD from three independent experiments), and a representative experiment is shown in the right panel. Phase contrast images were taken at × 4 magnification

To investigate the mechanism of this inhibition in CRC, we examined whether G9a, EZH2, and HDACs could influence the activity of the E-cadherin promoter. Specifically, we treated SW480-Snail2 cells transfected with the pGL3-E-cadherin-Luc vector with Bix-01294, GSK343, and TSA, to inhibit G9a, EZH2, and HDACs, respectively. We found that some of these drugs could inhibit the activity of E-cadherin promoter in SW480-Snail2 cell line (Fig. 4a). Upon treatment of cells overexpressing Snail2 with Bix-01294, the luciferase activity was still lower than in SW480-Snail2 cells, suggesting that G9a did not repress the E-cadherin promoter. On the contrary, upon treatment with GSK343 and TSA, the luciferase activity in Snail2-overexpressing cells significantly increased, suggesting that EZH2 and HDACs may repress the E-cadherin promoter. In addition, we examined the histone modification levels of the E-cadherin promoter using ChIP assays. We found that the level of H3K27me3 which was mainly catalyzed by EZH2 was significantly increased at the E-cadherin promoter after the overexpression of Snail2 in SW480 cell line (Fig. 4b). We also investigated the effect of specific HDACs in E-cadherin expression, and for this purpose, we used two inhibitors: LMK-235, which inhibits HDAC4 and HDAC5, and Tubastatin A, which inhibits HDAC6. As shown in Fig. 4c, upon treatment with Tubastatin A, the luciferase activity of Snail2-overexpressing cells increased, indicating that HDAC6 could represses the E-cadherin promoter. Furthermore, we found that Snail2 could specifically interact with HDAC6 in SW480 cells (Fig. 4d). However, the interaction between Snail2 and EZH2 was too weak to be detected (data not shown). In addition, HDAC6 could also bind with EZH2 in SW480 cells, suggesting that Snail2/HDAC2/EZH2 might form a functional complex to exert repression of the E-cadherin (Fig. 4d). Moreover, we treated SW480-Snail2 cells with EZH2 and HDAC6 inhibitors and the results showed that upon inhibitor treatment, the expression of epithelial markers including E-cadherin was upregulated, whereas mesenchymal markers such as fibronectin were downregulated (Fig. 4e, f). Taken together, our results fully supported the indispensability of EZH2 and HDAC6 in the Snail2-mediated E-cadherin repression.

To further demonstrate that EZH2 and HDACs (specifically, HDAC6) could influence the migration of colon cancer cells, Snail2-overexpressing cells were treated with GSK343, TSA, or Tubastatin A. As shown in Fig. 4g, compared with control cells (SW480-N), the migration of these cells significantly increased. In all, these data indicated that Snail2 may epigenetically repress E-cadherin expression.

## Discussion

Epigenetic alterations play a critical role in many human disorders [31]. Histone-modifying enzymes are aberrantly expressed in various cancers, leading to altered transcription states that change cell identity and behavior. Many studies have shown that acetylation, methylation, and phosphorylation are closely related to tumor invasion and metastasis. It has been reported that aberrant JMJD3 expression upregulates Snail2 to promote migration, invasion, and stem cell-like behaviors in hepatocellular carcinoma [32]. A previous study has found that MEF2D transduces microenvironmental stimuli to ZEB1 to promote EMT and metastasis in CRC [33]. Additionally, in bladder cancer, HIF-1α promotes ZEB1 expression and EMT. It is believed that CRC metastasizes easily, which might be the main cause of the high mortality associated with this cancer. Furthermore, some studies show the role of transcriptional inhibitors in EMT. However, little research has been conducted on the role of epigenetics in CRC.

A previous study has shown that concomitant high expression of Snail2 and low expression of E-cadherin in patients with CRC is associated with adverse prognosis, while patients with low expression Snail2 and high expression of E-cadherin have a favorable prognosis [34]. However, the function of Snail2 on the metastasis of CRC and the mechanism through which Snail2 represses E-cadherin expression is unclear. In our study, we first confirmed that Snail2 is highly expressed in patients with CRC. Meanwhile, the stable overexpression of Snail2 in SW480 cells promoted migration and invasion both in vitro and in a mouse model. The morphology of SW480-Snail2, together with the data relative to the expression of E-cadherin and vimentin, indicated EMT upon overexpression of Snail2.

Snail1 interacts with G9a, an important euchromatin methyltransferase responsible for the formation of H3K9me2, and recruits G9a and other DNA methyltransferases to the E-cadherin promoter for DNA methylation. Additionally, PRC2 plays an important role in differentiation, maintenance of cell identity, and proliferation [29, 35], and it is deregulated in a wide variety of cancers, in which it can have oncogenic or tumor-suppressive activity depending on context. However, little is known about the functions of PRC2 in the metastasis of CRC. On the other hand, HDACs promote chromatin condensation and, by repressing gene transcription, play a role in cell cycle regulation and cell differentiation [36]. Many studies have shown that HDACs are associated with the development of tumors.

Our study showed that Snail2 might recruit the histone deacetylases HDAC6 and histone methyltransferase PRC2 to the promoter of E-cadherin to inhibit its expression. Therefore, the overexpression of Snail2 promotes EMT and induces invasion and metastasis in CRC. Taken together, our findings identify a new mechanism of metastasis in CRC and open the way for additional research that might be instrumental in discovering novel ways to fight cancer.

## Conclusion

Snail2 is highly expressed in clinical specimens of colorectal cancer. In addition, overexpression of Snail2 induced migration and metastatic properties in CRC cells in vitro and in vivo. Furthermore, overexpression of Snail2 promoted the occurrence of the epithelial–mesenchymal transition, downregulating the expression of E-cadherin and upregulating that of vimentin. Specifically, Snail2 could interact with HDAC6 and then recruits HDAC6 and PCR2 to the promoter of E-cadherin and thus inhibit the expression of E-cadherin and induce invasion and metastasis of CRC. The overexpression of Snail2 promotes EMT and induces invasion and metastasis in CRC. Thus, our study reveals a critical mechanism underlying the epigenetic regulation of EMT by Snail2. Our findings identify a new mechanism of metastasis in CRC and open the way for additional research that might be instrumental in discovering novel ways to fight cancer.

## Abbreviations

CRC: Colorectal cancer
EGF: Epidermal growth factor
EMT: Epithelial–mesenchymal transition
EZH2: Enhancer of zeste homolog 2
HDAC6: Histone deacetylases6
HGF: Hepatocyte growth factor
JMJD3: Histone demethylase jumonji D3
PRC2: Polycomb repressive complex 2
TGF: Transforming growth factor
